# Comparative Study of Aromatic and Cycloaliphatic Isocyanate Effects on Physico-Chemical Properties of Bio-Based Polyurethane Acrylate Coatings

**DOI:** 10.3390/polym12071494

**Published:** 2020-07-03

**Authors:** Nurul Huda Mudri, Luqman Chuah Abdullah, Min Min Aung, Mek Zah Salleh, Dayang Radiah Awang Biak, Marwah Rayung

**Affiliations:** 1 Department of Chemical and Environmental Engineering, Faculty of Engineering, Universiti Putra Malaysia, Serdang 43400, Selangor, Malaysia; dradiah@upm.edu.my; 2 Radiation Processing Technology Division, Malaysian Nuclear Agency, Kajang 43000, Selangor, Malaysia; mekzah@nm.gov.my; 3 Institute of Tropical Forestry and Forest Products (INTROP), Universiti Putra Malaysia, Serdang 43400, Selangor, Malaysia; minmin_aung@upm.edu.my (M.M.A.); marwahrayung@yahoo.com (M.R.); 4 Centre of Foundation Studies for Agricultural Science, Universiti Putra Malaysia, Serdang 43400, Selangor, Malaysia; 5 Institute of Advanced Technology, Universiti Putra Malaysia, Serdang 43000, Selangor, Malaysia; 6 Department of Chemistry, Faculty of Science and Technology, Universiti Putra Malaysia, Serdang 43400, Selangor, Malaysia

**Keywords:** jatropha oil, polyurethane acrylate, 2,4-toluene diisocyanate (2,4-TDI), isophorone diisocyanate (IPDI), UV curing

## Abstract

Crude jatropha oil (JO) was modified to form jatropha oil-based polyol (JOL) via two steps in a chemical reaction known as epoxidation and hydroxylation. JOL was then reacted with isocyanates to produce JO-based polyurethane resin. In this study, two types of isocyanates, 2,4-toluene diisocyanate (2,4-TDI) and isophorone diisocyanate (IPDI) were introduced to produce JPUA-TDI and JPUA-IPDI respectively. 2,4-TDI is categorised as an aromatic isocyanate whilst IPDI is known as a cycloaliphatic isocyanate. Both JPUA-TDI and JPUA-IPDI were then end-capped by the acrylate functional group of 2-hydroxyethyl methacrylate (HEMA). The effects of that isocyanate structure were investigated for their physico, chemical and thermal properties. The changes of the functional groups during each synthesis step were monitored by FTIR analysis. The appearance of urethane peaks was observed at 1532 cm^−1^, 1718 cm^−1^ and 3369 cm^−1^ while acrylate peaks were detected at 815 cm^−1^ and 1663 cm^−1^ indicating that JPUA was successfully synthesised. It was found that the molar mass of JPUA-TDI was doubled compared to JPUA-IPDI. Each resin showed a similar degradation pattern analysed by thermal gravimetric analysis (TGA). For the mechanical properties, the JPUA-IPDI-based coating formulation exhibited a higher hardness value but poor adhesion compared to the JPUA-TDI-based coating formulation. Both types of jatropha-based polyurethane acrylate may potentially be used in an ultraviolet (UV) curing system specifically for clear coat surface applications to replace dependency on petroleum-based chemicals.

## 1. Introduction

Polyurethane (PUR) is a versatile resin that can be used for various applications such as coatings, adhesives, sealants and elastomers. Basically, PUR is synthesised by reaction of polyols with isocyanates at a specific controlled temperature and reaction speed in a nitrogen purged system. Currently, most of the polyols that are used in synthesising PUR are derived from petroleum and natural gas sources [1,2]. As non-renewable sources, petrochemical resins are facing issues with supply shortages and worldwide price fluctuations. Moreover, the processing of these chemicals may release greenhouse gases that are harmful to the environment, thus leading to global warming. Alternatively, usage of low toxic natural-based polymeric materials has been proposed to replace the dependency on petroleum-based chemicals in order to achieve sustainable development [3,4].

Vegetable oil, cellulose and sugar are among the bio-based feedstock that have been used in producing PUR [1,5]. Between these feedstocks, vegetable oil serves as the most promising option to function as the polyol starting material because of its availability, simple processing method and being cost effective. Moreover, it has a unique characteristic whereby the unsaturated part of the backbone of the triglycerides enables it to undergo chemical modification [3,4]. Several bio-based oil such as soy [5], palm [6], castor [2], canola [2], neem [7], etc., have been studied for PU application. However, these vegetable oils are classified as edible oils and the utilisation of food supplies for a non-food purpose has attracted a negative perception from society.

The *Jatropha curcas* tree is one of the main industrial crops in Malaysia with a total coverage area of 809 ha [8]. It is traditionally used in soap, fertilisers and as an alternative medicine by the local community. The oil is extracted from the seed of the fruit of jatropha with a yield of around 30–40% of oil content. It has a high unsaturated fatty acid content (78–84%) given by oleic acid (C18:1) and linoleic acid (C18:2) which reflects its sensitivity to chemical reaction [9]. Jatropha oil (JO) is a non-edible oil because of its phorbic ester compound that is toxic for oral consumption [10]. Therefore, converting jatropha oil into polyols for the purpose of PU production has no critic related to the edible oil source issue.

Previous researchers have investigated the optimum parameters to synthesis jatropha oil-based epoxy [11,12] and polyols resin [13] for coating applications. Several studies have been conducted related to Jatropha oil-based polyurethane acrylate (JPUA) but only limited to Toluene diisocyanates (TDI)-based isocyanates for different applications such as wood coating [14], and a polymer electrolyte [15,16].

A few of isocyanates are commonly used in the coating industry such as 2,4-TDI, 4,4′-methylenediphenyl diisocyanate (MDI), 1,6-hexamethylene diisocyanate (HDI), and isophorone diisocyanate (IPDI). Among these isocyanates, TDI is the most reactive because of the existence of its aromatic ring [17]. Diisocyanate is a volatile compound that may affect human health via the inhalation route. Studies of cancer risk have revealed that TDI and MDI are linked to cancer risk with the ability to bind the DNA and lead to genotoxicity [18]. Meanwhile, HDI and IPDI are safer and have been testified as not associated with carcinogenic effects [19,20].

In this study, a jatropha oil-based polyurethane (JPUA) was synthesised based on the reaction between jatropha oil-based polyol (JOL) with 2,4-TDI and IPDI. The effects of the linear chain and aromatic ring of the isocyanates were investigated related to the physicochemical properties of the JPUA coating.

## 2. Materials and Methods

### 2.1. Materials

Crude jatropha oil was purchased from Biofuel Bionas Malaysia Sdn. Bhd., Malaysia. Hydrogen peroxide (30%), formic acid (98%), sulfuric acid (95%) and methanol (99.8%) were supplied by R&M Chemicals, India. 2,4-TDI (IUPAC name: 2,4-Diisocyanato-1-methylbenzene) (95%), IPDI (IUPAC name: 5-isocyanato-1-(isocyanatomethyl)-1,3,3-trimethylcyclohexane) (98%), dibutyltin dilaurate (IUPAC name: [dibutyl(dodecanoyloxy)stannyl] dodecanoate) (DBTDL) (95%), HEMA (IUPAC name: 2-Hydroxyethyl 2-methylprop-2-enoate) and trimethylolpropane triacrylate (TMPTA) were supplied by Sigma-Aldrich, Germany. *N,N*-Dimethylformamide (DMF) (98%) and benzophenone (IUPAC name: Diphenylmethanone) were purchased from Fisher Scientific (USA) and Acros Organic (Belgium) respectively. All chemicals were used as supplied without further purification.

### 2.2. Synthesis of JOL

The bio-based JOL was prepared in two steps namely epoxidation and hydroxylation. This method was adopted from [13] with slight modification. For epoxidation, the reaction was performed based on a molar ratio of 1:0.6:1.7 for JO: formic acid: hydrogen peroxide. This experimental set-up was designed based on an amount of 400 g of JO per batch. 

Initially, JO was mixed with formic acid at 300 rpm at 40 °C until it became homogenous. Then, hydrogen peroxide was added slowly using a dropping funnel and the temperature was raised to 60 °C. In each hour, an oxygen oxirane content test (OOC) was performed based on the ASTM D1652-97 Method A standard. The reaction continued for 4.5 h based on previous literature in order to achieve the maximum value of OOC. The mixture was then cooled and transferred into a separating funnel. The aqueous layer was discarded and the epoxidised jatropha oil (EJO) was washed with distilled water until it achieved a neutral pH level.

In the second reaction, a stoichiometric of EJO (10 mol) was reacted with a mixture of methanol (9 mol) and 0.07% diluted sulfuric acid (1 mol). At first, methanol in the presence of the acid catalyst was activated by a heating and stirring process under conditions of 40 °C, 300 rpm for 15 min. Afterwards, the EJO was added into the solution and continuously mixed for 30 min at 65 °C. OOC was conducted to check the disappearance of the oxirane ring during the synthesis process by the titration method. The JOL mixture was cooled and then moved into a separating funnel to remove the aqueous layer. The JOL was washed with distilled water until it was free from acid. The excess water and methanol were removed using a rotary evaporator at a temperature of 60 °C. The JOL was then tested for hydroxyl value (OHV) referring to ASTM D4274-99 Test Method C prior to the isocyanation reaction.

### 2.3. Synthesis of JPUA

A corresponding amount of JOL: 2,4-TDI: HEMA was calculated based on a molar ratio of 1:1:1. The reaction was accomplished in a four-neck flask complete with a stirrer and condenser in a nitrogen purged system. First, JOL was mixed with DMF at 60 °C with a stirring rate of 350 rpm. Next, 2,4-TDI was added in a dropwise manner within 15 min and the reaction was mixed continuously for 2 h. Then, the temperature was cooled down to 40 °C and HEMA was added into the mixture. Later, the temperature was raised up to 70 °C for 1 h upon complete termination of the reaction of JPUA-TDI. During the reaction, DMF was added into the mixture to control the viscosity of the solution to avoid the gelling phenomena. The amount of DMF was set to be 50% (*w*/*w*) of the total amount of JPUA-TDI.

The same procedure was repeated for the IPDI-based JPUA with the same NCO/OH ratio. However, 1% (*w*/*w*) of DBTDL was added into the mixture of JOL as a catalyst to initiate the reaction with the IPDI-based isocyanate.

### 2.4. Formulation and Curing of JPUA

In the JPUA-TDI and JPUA-IPDI-coating formulation, TMPTA and benzophenone were selected as a trifunctional acrylate monomer and photoinitiator respectively. The quantity of benzophenone was set at 4% (*w*/*w*) of the total amount of the formulation. The amount of each component of the formulation is listed in Table 1 and Table 2 respectively.

### 2.5. Preparation of UV-Cured Film

The coating formulation was coated on a glass substrate (10 cm × 10 cm × 0.3 cm) using a bar applicator with a thickness of 50 µm. The wet film was exposed under UV radiation (UV-IST, Nürtingen, Germany) at an energy level of 200 W/cm at a speed of 5 m/min. The film was considered fully cured after nine passes under UV light based on non-tackiness test using a finger (ASTM D1640).

### 2.6. Characterisation

#### 2.6.1. Viscosity

The viscosity of the JO, EJO, JOL, JPUA-TDI and JPUA-IPDI were determined by using a Haake Mars Rheometer (Thermo Scientific, Waltham, MA, USA) at 25 °C. Approximately 1 g of resin was placed on the sample platform for testing. A flat plate spindle (PP 60) was moved on the sample in rotation mode with a gap of 1 mm. The viscosity was measured using plate-plate geometry mode and analysed by Rheo Win software.

#### 2.6.2. Tintometer

The colour measurement was performed using a Lovibond Colour Tintometer (Model F, Amesbury, UK). The colour recognition was achieved based on matching the similarity of the resin colour with the combination colour filter of red, yellow, blue and neutral.

#### 2.6.3. Fourier Transform Infra-Red (FTIR) Spectroscopy

The conversion of the functional group during the chemical reactions was analysed using FTIR (Bruker Tensor II, Ettlingen, Germany). It was equipped with an attenuated total reflectance (ATR) accessory with a pure diamond crystal. The reading was in the spectral range of 500–4000 cm^−1^ with a resolution of 4 cm^−1^. This instrument ran an average of 32 scans per sample.

#### 2.6.4. Gel Permeation Chromatography (GPC)

The molar mass of the polymer and its polydispersity index (PDI) were determined by using GPC (Waters Corporation, MA, USA). Total of 5 mg of sample was diluted in 5 mL THF and injected into three types of column; Styragel HR1, Styragel HR 3 and Styragel HR 5 respectively.

#### 2.6.5. Thermal Analysis

Thermal gravimetric analysis (TGA) of the JPUA-TDI and JPUA-IPDI-cured film was conducted using a Netzsch (TG209 F3, Selb, Germany) tester based on ASTM E1131. The samples were run under conditions from room temperature up to 700 °C with a heating rate of 10 °C/min under a nitrogen-purged atmosphere.

#### 2.6.6. Pendulum Hardness

The hardness of the UV-cured film was measured using a Pendulum Hardness tester (TQC, Capelle aan den Ijssel, The Netherlands). The test was conducted referring to ASTM D4366 with the König damping mode. The data was counted in seconds during the damping time of the pendulum till it totally stopped. Readings were taken in triplicate for each sample and the average value was calculated.

#### 2.6.7. Adhesion Test

A cross hatch adhesion test was done according to ASTM D3359-09 (Biuged, Guangzhou, China). The kit came complete with a cutter blade, tape, brush and magnifying glass. A 1-mm space cutter blade was chosen based on the coating thickness to prepare the lattice pattern. A piece of 3M Scotch transparent tape was applied on the lattice and pulled off at approximately at a 180° angle. Any dirt was gently removed using the brush and the surface was examined using a magnifying glass. The adhesion score was given based on the removed area of the lattice as described in Table 3.

#### 2.6.8. Contact Angle

Static contact angles were examined on the surface of the cured film of JPUA-TDI and JPUA IPDI at 25 °C using an Attension Theta Optical Contact Angle tester (Biolin Scientific, Manchester, UK). Approximately 7 µL of deionised water was applied on the coating surface using the sessile dropping method. The contact angle readings were measured based on its intersection with the surface within one minute after the deionised water was dropped.

#### 2.6.9. Transmittance and Haze Tests

The transmittance (ASTM E1348) and haze tests (ASTM D1003) of the JPUA-TDI and JPUA-IPDI cured film were performed using a Haze Illuminant tester (BYK-Gardner, Geretsried, Germany). This machine measured both properties simultaneously as a percentage value. All readings were taken three times and the average value was recorded.

## 3. Results and Discussion

### 3.1. Synthesis of JPUA

Two series of JPUA namely JPUA-TDI and JPUA-IPDI were successfully synthesised. The physical properties of the products during stepwise synthesis reaction are reported in Table 4.

Colour measurement is one of the physical properties for oil and its derivatives. Any colour changes provide information on production output during physical treatment and chemical reaction. In this study, the colour of the JO-based resin differed in each reaction step. The colour of JO in its original form was brownish and then changed to light yellow and yellow after reaction of epoxidation and hydroxylation. For the isocyanation stage, the colour of JPUA-TDI was slightly brownish while JPUA-IPDI was visually seen as light yellow. This observation was comparable as reported by [15] for JPUA-TDI. The colour difference between JPUA-TDI and JPUA-IPDI may affect the aesthetic property of the end-product [21]. To avoid human bias during visual assessment, specific colour recognition was conducted using a tintometer as listed in Table 4. Under room conditions, all resins were in liquid form except JPUA-TDI which was in a viscous semi-liquid form.

### 3.2. FTIR Analysis

**Figure 1:**
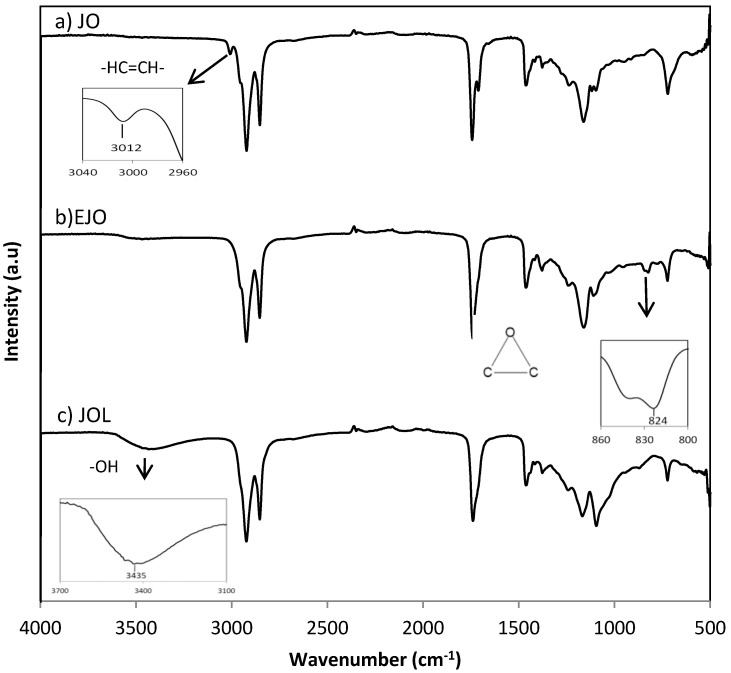
Fourier transform infra-red (FTIR) spectra during epoxidation and hydroxylation of jatropha oil.

**Figure 2:**
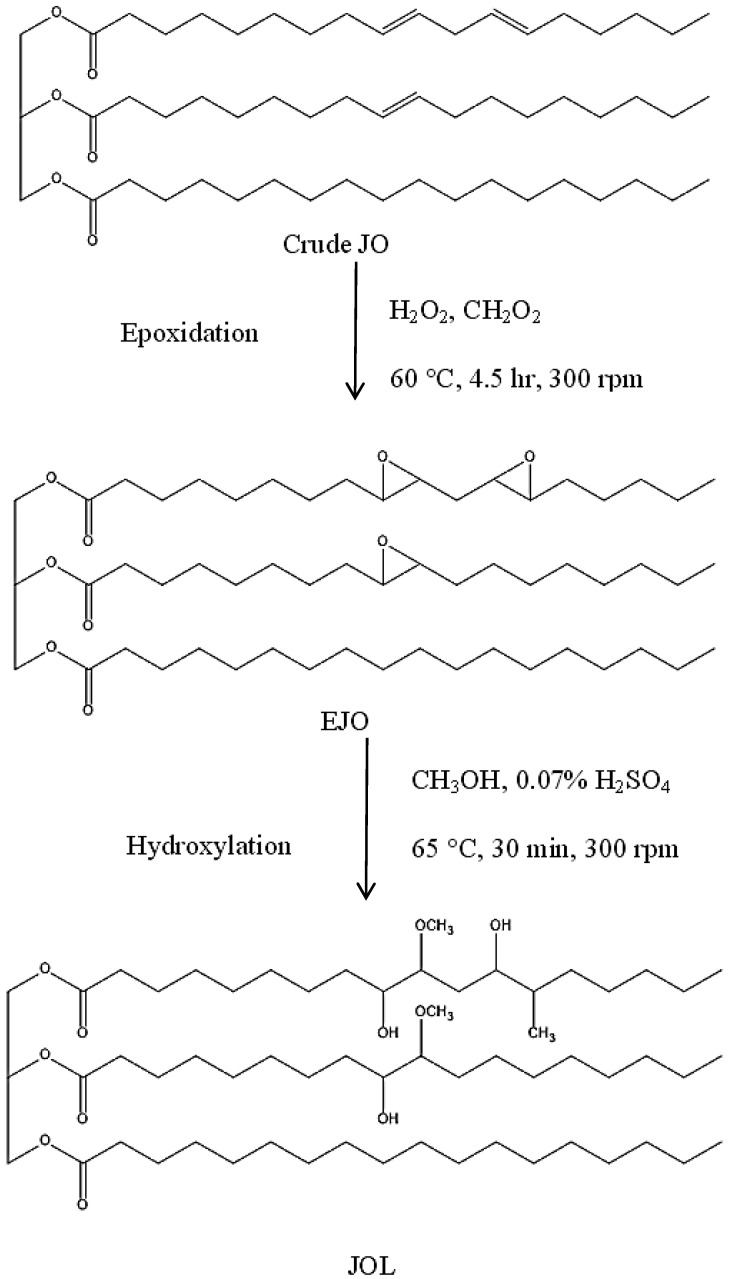
Proposed chemical reactions to produce jatropha oil-based polyol (JOL).

The comparative FTIR spectra in preparation of natural-based polyols of jatropha oil is shown in Figure 1. In its original state, a signal of the carbon double bond of the crude JO was detected at 3012 cm^−1^. A similar trend was observed in the other types of natural oil such as palm [22] and canola oil [2]. This peak then disappeared during the epoxidation reaction where the carbon double bond was converted into an oxirane ring (C-O-C) upon reaction with hydrogen peroxide which was observed at 824 cm^−1^. Based on the titration method, the OOC obtained was 4.25 ± 0.08% (Table 4). Next, the oxirane ring broke during hydroxylation which was indicated by the disappearance of the oxirane peak (824 cm^−1^) and a new broad peak of the OH group was formed at 3435 cm^−1^. The OHV achieved for JOL was 149.44 ± 0.23 mg KOH/g (Table 4). The predicted stepwise chemical reactions of JOL are depicted in Figure 2.

**Figure 3:**
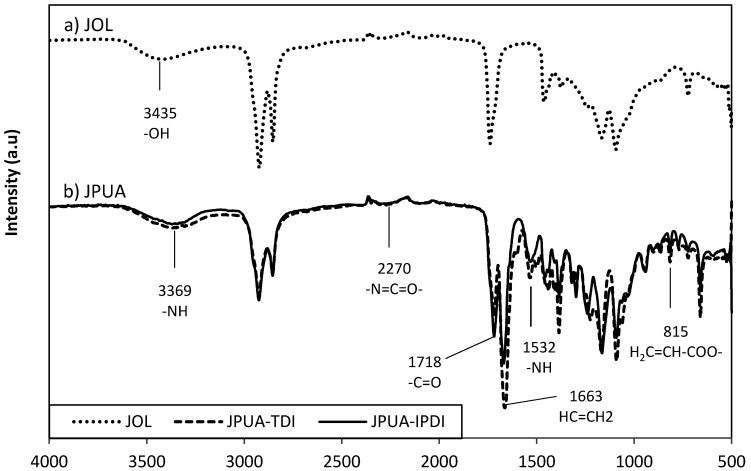
Conversion of (**a**) JOL into (**b**) JPUA-TDI and JPUA-IPDI via the isocyanation reaction.

The FTIR spectrum during isocyanation of JOL is depicted in Figure 3. The absorption peak at 1532 cm^−1^ and 1718 cm^−1^ corresponded to amide (-NH) bending and amide (-NH) stretching respectively. The peak of carbonyl (C=O) was observed at 3369 cm^−1^ [23]. The absence of the (N=C=O) peak reflected that all isocyanates either from 2,4-TDI or IPDI were consumed during the reaction [15,16]. The characteristics of the acrylate group were determined by the absorption band of 1663 and 815 cm^−1^ which related to the acrylate double bond (-CH=CH_2_) and vinyl functionality of (CH_2_=CH-COO-) respectively [24]. Both JPUA-TDI and JPUA-IPDI indicated a similar trend under FTIR because of possessing the same functional groups.

Generally, the reactivity of the reaction depends on the structure of isocyanate being used. The electrophilic carbon of isocyanate (N = **C** = O) was attacked by nucleophilic of oxygen (**O**-H) from alcohol during the isocyanation process. In 2,4-TDI, the benzene ring acted as an electron withdrawing group via an inductive effect. The negative charge in benzene was then delocalised in the π ring system. This enhanced the positive charge on the carbon of isocyanate (N = **C** = O) to further undergo a nucleophilic addition reaction with oxygen (**O**-H) derived from JOL. Therefore, this raised the reactivity and speeded up the reaction during isocyanation [25].

**Figure 4:**
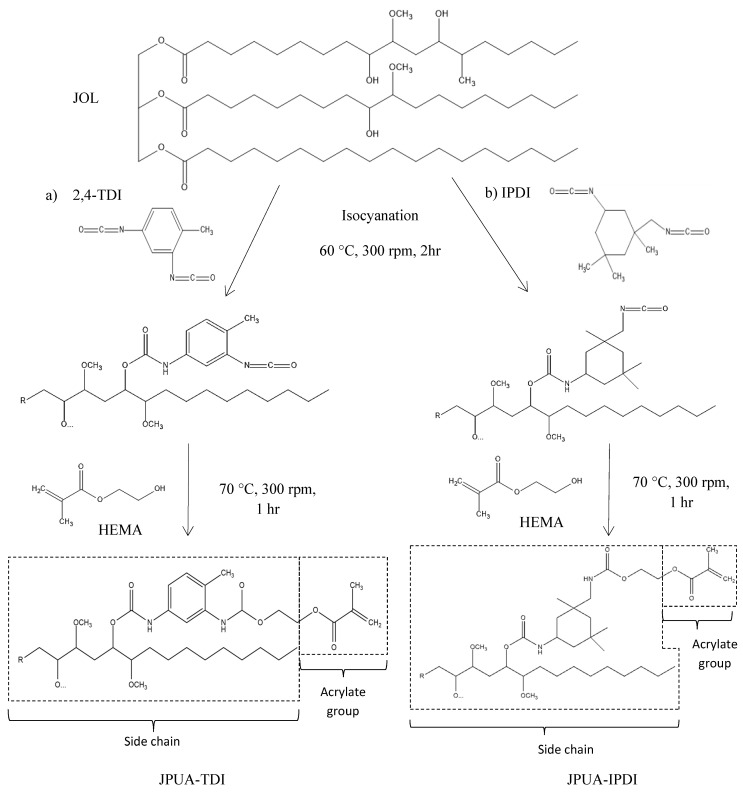
Isocyanation of JOL to produce (**a**) JPUA-TDI and (**b**) JPUA-IPDI.

Apart from that, the steric hindrance effect formed in IPDI because of the existence of three methylene (-CH_3_) groups which bonded to the cyclic ring. This effect reduced the reactivity of the IPDI and slowed down the isocyanation reaction [25]. The chemical structures explain the reason for the high reactivity of 2,4-TDI compared to IPDI. The proposed chemical reactions of JPUA-TDI and JPUA-IPDI are illustrated in Figure 4.

### 3.3. Viscoelasticity Property

The molar mass and viscosity of all the resins in this study are presented in Table 5. It can be observed that the molar mass of the JO-based products increased at each stage of the synthesis. This indicated that additional chains were formed via grafting of functional groups such as epoxy, hydroxyl and isocyanates onto the triglyceride backbone. The polydispersity index (PDI) of all jatropha oil-based resins except JPUA-TDI was near to 1.00. This reflected a good distribution of the molar mass within a narrow bell shape curve [26].

For the viscosity property, the higher crosslinking in each reaction step reflected the longer polymer chain which led to higher viscosity [6]. This trend agreed with the current finding for JO, EJO and JOL. However, the trend slightly changed for JPUA-TDI and JPUA-IPDI because of a calculated amount of 50% (*w*/*w*) DMF added during the isocyanation reaction to avoid gelation. The additional solvent decreased the viscosity of the JPUA-TDI and JPUA-IPDI from their actual values.

Nevertheless, to compare the viscosity only between JPUA-TDI and JPUA-IPDI resins would be considered valid as the success of the reaction was supported by FTIR and GPC data. From Table 5, the viscosity value of JPUA-TDI was far higher (10.82 Pas) compared to JPUA-IPDI (0.095 Pas). The fast reaction between the reactive 2,4-TDI with JOL produced a bulk molar mass (Mw) compared to the average molecular number (Mn). This led to a broad distribution of molar mass (PDI = 2.15) with a high viscosity JPUA-TDI resin. Thus, JPUA-TDI required an appropriate reactive diluent to achieve the intended viscosity during the coating process. For JPUA-IPDI, a good distribution of molecular (PDI = 1.28) may maintain low viscosity resin [26].

### 3.4. Thermal Analysis

**Figure 5:**
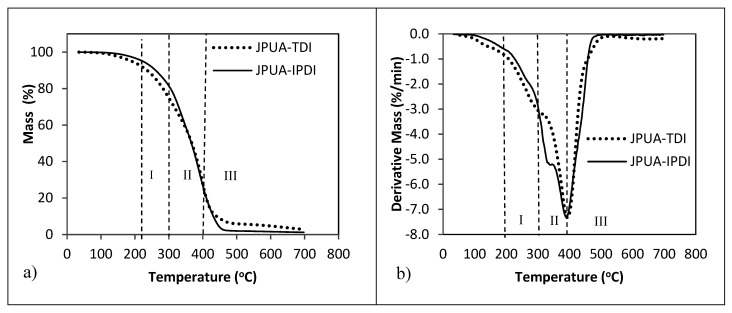
(**a**) Thermal gravimetric analysis (TGA) thermogram and (**b**) derivative curve of JPUA-TDI and JPUA-IPDI.

TGA analysis is useful in providing information regarding the purity of a compound and its stability temperature during processing. The TGA thermogram and weight loss derivative curves in Figure 5 revealed that both the JPUA series were degraded in three stages. At temperatures below 100 °C, trapped water was removed during the degradation process. The first stage was observed in the range of 200–300 °C. This 18–25% weight loss corresponded to the decomposition of urethane linkage into primary or secondary amine, olefin and dioxide. The second stage of degradation occurred between 300–400 °C which was attributed to the cleavage of the backbone of the oil mainly from the aliphatic and alkyl groups of the fatty acid. Major degradation took place at this temperature range with a weight loss of up to 73%. Finally, the thermal degradation beyond a temperature of 400 °C was contributed by further thermo-oxidation of the JPUA films. These findings were comparable with [27,28] for other types of polyurethane-based coatings. Even though JPUA-TDI (6871 g/mol) had a higher molar mass compared to JPUA-IPDI (3151 g/mol), both showed a similar thermal stability pattern with a final residue of 2.8% and 1.2% respectively.

### 3.5. UV Curing of the Coating

**Figure 6:**
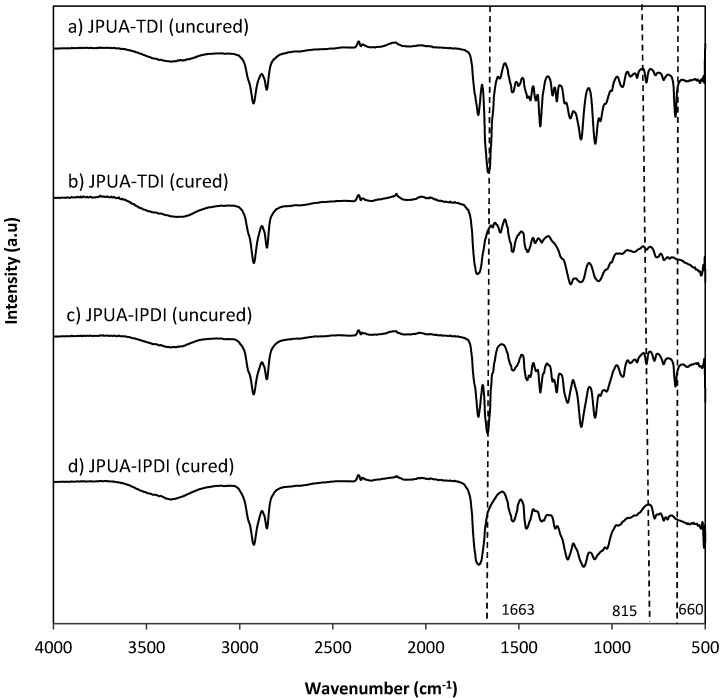
FTIR curves of JPUA-TDI and JPUA-IPDI before and after UV irradiation respectively.

In a UV curing system, acrylate is the functional group that is responsible to react with radical species during polymerisation. The radical species was generated by a photoinitiator after being exposed to UV light. Based on the FTIR spectra in Figure 6, the acrylate absorption peak at 1663 cm^−1^ (-CH=CH_2_), 815 cm^−1^ (CH_2_=CH-COO-) 660 cm^−1^ (C=C bending) was wiped out after exposure under UV irradiation. This data indicated that the acrylate group had been utilised during the radical polymerisation reaction. This finding matched with other types of acrylate system such as epoxidised soy bean acrylate [29], epoxidised palm acrylate [22], epoxidised Jatropha acrylate [24] and a waterborne acrylate system [28].

### 3.6. Mechanical Properties of Cured Film

The mechanical property tests were conducted on a cured film containing either JPUA-TDI or JPUA-IPDI with corresponding ratio of a TMPTA monomer and benzophenone as a photoinitiator. Initially, benzophenone induced the curing reaction by absorbing the energy from the UV light and became excited to a higher energy level. Next, benzophenone became unstable and experienced a photocleavage reaction and turned into a radical species known as benzoyl radical. Benzoyl radical attacked the carbon double bond (C = C) on the acrylate part which was given by JPUA-TDI, JPUA-IPDI (Figure 3) and TMPTA for propagation of the polymer chain. This reaction continued until all the acrylate was used up, thus reaching the termination reaction of the polymerisation [30].

#### 3.6.1. Hardness Test

**Figure 7:**
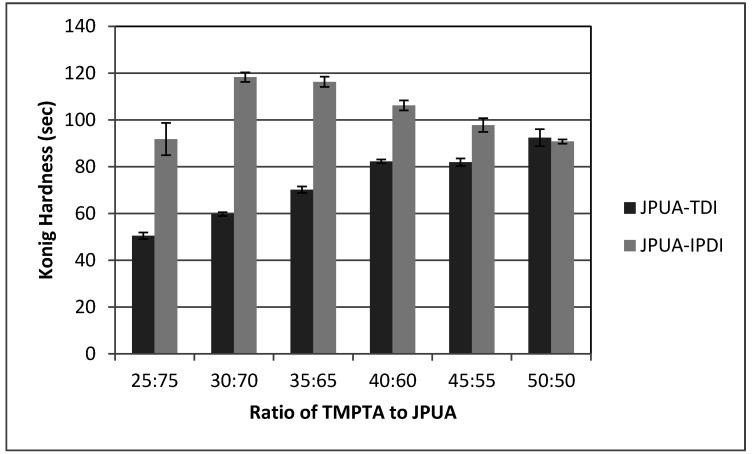
Hardness measurement of JPUA-TDI and JPUA IPDI-based coating.

The hardness of JPUA-TDI and JPUA-IPDI films is presented in Figure 7. The hardness property reflects the coating toughness and its durability. From the result, JPUA-IPDI had a higher hardness value compared to JPUA-TDI-based film. The hardness characteristic was linked with crosslinking density that was highly dependent on the JPUA-TDI and JPUA-IPDI molecular structure as illustrated in Figure 4.

The symmetrical rigid structure of the side chain of 2,4-TDI on the JPUA-TDI backbone caused steric hindrance. This effect generated constraints for the benzoyl radical to attack the carbon double bond (C = C) of the acrylate chain during the propagation state of radical polymerisation. Meanwhile, JPUA-IPDI offered a more flexible, asymmetrical aliphatic structure of IPDI side chain with less steric hindrance [31]. This made JPUA-IPDI more favourable during the UV curing reaction compared to JPUA-TDI. 

The hardness property of the film increased as the ratio of monomer increased because of the high crosslinking polymerisation via hydrogen bonding [6,22] between JPUA and TMPTA. In the case of JPUA-IPDI, the hardness dropped gradually starting at a ratio of 40:60 because of maximum limit of the reactive site of polymerisation as the trifunctional acrylate continued to be added. This may have caused the degradation of chain scission of the polymer network as well as leading to the decrease of the hardness value.

#### 3.6.2. Adhesion Test

The chemical structure of the polymer and its flexibility highly affects the adhesion property. From the result in Table 6, the increasing amount of trifunctional acrylate given by TMPTA led to a higher degree of crosslinking network after being cured under UV radiation. A high density of crosslinking caused the surface to become brittle [6,22].

The JPUA-TDI based film had a better adhesion property than the JPUA-IPDI which matched its lower hardness value (Section 3.6.2). This indicated that JPUA-TDI had a lower density crosslinking network and inversely owned good flexibility compared to JPUA-IPDI. The addition of TMPTA in both the JPUA-TDI and JPUA-IPDI-based resins of more than 35% caused a brittle film surface and reduced bonded attachment to the substrate. This resulted in a decreased adhesion score phenomena in JPUA-TDI from 4B to 1B and 0B. JPUA-IPDI also showed a similar trend with declining adhesion performance from 1B to 0B.

#### 3.6.3. Water Contact Angle

The wettability of JPUA-TDI and JPUA-IPDI-based coating were investigated using the water contact angle as presented in Table 7. It was found that all coating formulations were in the range of 75–89° which was nearly hydrophobic (90°). A hydrophobic surface indicates that it can prevent itself from contamination by any moisture condition and increases the lifetime of the substrate especially for metal and wood [32].

The crosslinking density and flexibility of the molecular chain also affected the surface wettability property [33,34]. The JPUA-TDI-based coating showed an increasing contact angle value from 75.84° to 87.13°. This trend was proportional to the addition of the TMPTA monomer ratio. Meanwhile, the JPUA-IPDI-based coating displayed an inverse trend of contact angle value from 88.35° to 79.87° when the percentage of TMPTA added into the coating formulation was increased. The higher crosslinking density in the JPUA-IPDI-based coating tended to increase the surface energy and its interfacial tension. Consequently, it decreased the contact angle value. Contrariwise, the steric hindrance effect led to a reduced crosslinking network in the JPUA-TDI-based coating. This contributed to more chain mobility in the JPUA-TDI-based coating structure and directed to low surface energy. Hence, the trend of the contact angle value in the JPUA-TDI-based coating increased with the increasing amount of TMPTA. This result was comparable with [33] who investigated the influence of reactive diluent on the water contact angle property.

#### 3.6.4. Transmittance Test

**Figure 8:**
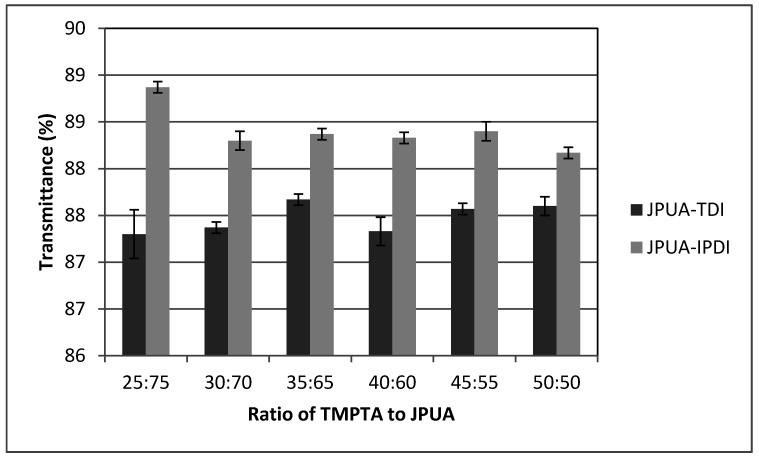
Transmittance value for variation of JPUA-TDI and JPUA-IPDI films.

The optical property of the films was characterised by a Transmittance and Haze test. The transmittance measures the amount of light that passes through a material. Based on Figure 8, all JPUA-TDI and JPUA-IPDI-based coating formulations were categorised as transparent materials as the transmittance values were maintained above 85% [35]. The incorporation of TMPTA in the formulation did not show any trend in terms of transmittance value for both the JPUA-TDI and JPUA-IPDI-based coatings.

#### 3.6.5. Haze Test

Haze is defined as the cloudy appearance of a material because of the light scattering of an imperfect surface. Several factors have been identified to affect the haze property such as poor dispersion, the coating technique, curing method, types of materials and additives and surface treatment.

**Figure 9:**
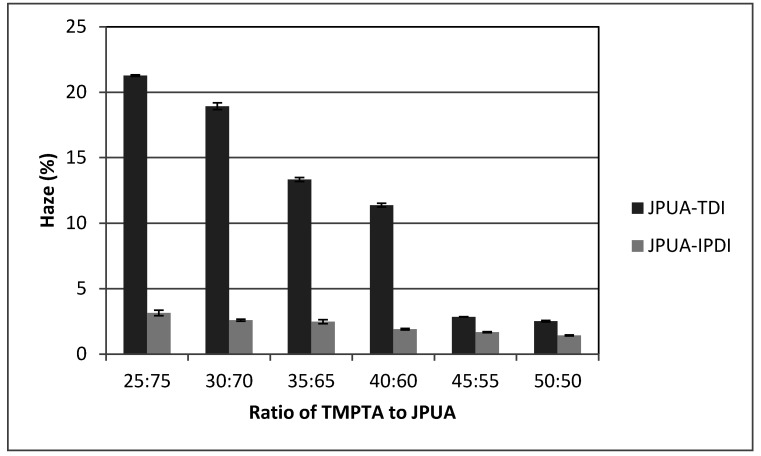
Haze data of JPUA-TDI and JPUA-IPDI-based films.

From Figure 9, JPUA-IPDI maintained a low haze for all TMPTA ratios with haze values of around 3% which complies with the standard for clear materials in industry. However, JPUA-TDI at an initial ratio of 25:75 showed a higher haze value of 22.3%. The increasing amount of TMPTA as a reactant diluent into the JPUA-TDI coating formulation improved its viscosity. Hence, a good dispersion of the coating formulation reduced the haze value of the JPUA-TDI-based film [36]. The JPUA-TDI-based film started to achieve the industry requirement range at ratio of 45:55 at a haze reading of 2.81 ± 0.01%.

## 4. Conclusions

Two varieties of JPUA based on TDI and IPDI isocyanates have been successfully synthesised. JPUA-TDI had a higher viscosity and molar mass with a broad PDI value compared to JPUA-IPDI resin. Both JPUA-TDI and JPUA-IPDI showed a similar degradation trend under TGA analysis. The JPUA-TDI-based formulation had low hardness but good adhesion compared to the JPUA-IPDI-based formulation. The best adhesion for both JPUA-TDI and JPUA-TDI-based films were at a ratio of 35:65. Both JPUA-TDI and JPUA-IPDI coating formulations owned a good transparency property. It is suggested that both JPUA-TDI and JPUA-IPDI can be used for clear coat coatings such as for windows and for the automotive and construction industry.

## Figures and Tables

**Table 1:** Jatropha oil-based polyurethane-toluene diisocyanate (JPUA-TDI)-based coating formulation.

Code	TMPTA (%)	JPUA-TDI (%)	Benzophenone (%)
TDI 25	25	75	4
TDI 30	30	70	4
TDI 35	35	65	4
TDI 40	40	60	4
TDI 45	45	45	4
TDI 50	50	50	4

**Table 2:** Components of jatropha oil-based polyurethane-isophorone diisocyanate (JPUA-IPDI)-based coating.

Code	TMPTA (%)	JPUA-IPDI (%)	Benzophenone (%)
IPDI 25	25	75	4
IPDI 30	30	70	4
IPDI 35	35	65	4
IPDI 40	40	60	4
IPDI 45	45	45	4
IPDI 50	50	50	4

**Table 3:** Explanation of adhesion grading.

Grade	Removed Area (%)	Appearance
5B	None	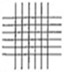
4B	Less than 5%	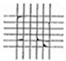
3B	5–15%	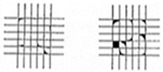
2B	15–35%	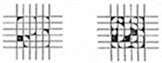
1B	35–65%	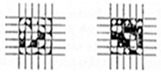
0B	Greater than 65%	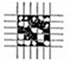

**Table 4:** Properties of jatropha oil-based resins intermediate to produced JPUA.

Sample	Chemical Reaction	Appearance	Tintometer	State at Room Temp	OOC (% per mole)	OHV (mg KOH/g)
JO	No reaction	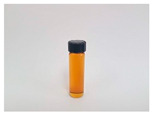	Red: 3.9 Yellow: 1.0 Blue: 0 Neutral: 0.2	Liquid	-	-
EJO	Epoxidation	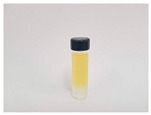	Red: 1.2 Yellow: 4.4 Blue: 0 Neutral: 0	Liquid	4.25 ± 0.08	-
JOL	Hydroxylation	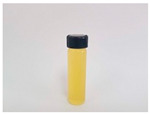	Red: 2.2 Yellow: 9.0 Blue: 0 Neutral: 0	Liquid	0.2 ± 0.03	149.44 ± 0.23
JPUA-TDI	Isocyanation (aromatic)	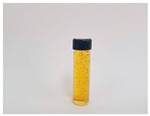	Red: 2.9 Yellow: 0 Blue: 0 Neutral: 2.0	Semi liquid	-	-
JPUA-IPDI	Isocyanation (cycloaliphatic)	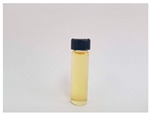	Red: 1.6 Yellow: 0 Blue: 0 Neutral: 3.0	Liquid	-	-

**Table 5:** Molar mass of jatropha-oil based resin.

Sample	Mw	Mn	PDI (Mw/Mn)	Viscosity (Pas)
JO	1278	948	1.35	0.056
EJO	1452	1041	1.39	0.342
JOL	1768	1426	1.23	2.336
JPUA-TDI	6871	3192	2.15	10.820
JPUA-IPDI	3151	2457	1.28	0.096

**Table 6:** Adhesion score of JPUA films.

JPUA Resin	Sample	Adhesion Score
**JPUA-TDI**	TDI 25	4B
	TDI 30	4B
	TDI 35	4B
	TDI 40	1B
	TDI 45	1B
	TDI 50	0B
**JPUA-IPDI**	IPDI 25	1B
	IPDI 30	1B
	IPDI 35	1B
	IPDI 40	0B
	IPDI 45	0B
	IPDI 50	0B

**Table 7:** Contact angle measurement of JPUA-TDI and JPUA-IPDI-based coatings.

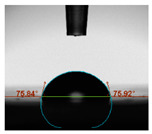	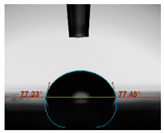	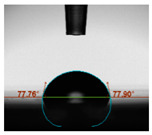	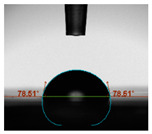	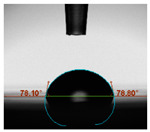	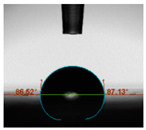
TDI 25	TDI 30	TDI 35	TDI 40	TDI 45	TDI 50
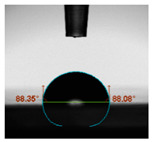	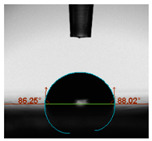	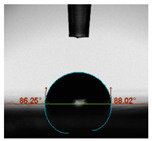	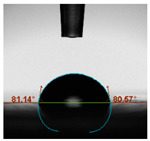	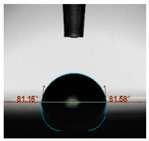	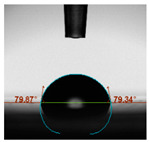
IPDI 25	IPDI 30	IPDI 35	IPDI 40	IPDI 45	IPDI 50
